# Extrarenal nephroblastomatosis in children: a report of two cases

**DOI:** 10.1186/1471-2431-14-255

**Published:** 2014-10-06

**Authors:** Yi Wu, Xueming Zhu, Xingdong Wang, Hangzhou Wang, Xu Cao, Jian Wang

**Affiliations:** Department of Pathology, Soochow University Affiliated Children’s Hospital, Jiangsu, 215003 China; Department of Pediatric Surgery, Soochow University Affiliated Children’s Hospital, Jiangsu, 215003 China; Institute of Pediatric Research, Soochow University Affiliated Children’s Hospital, Jiangsu, 215003 China

**Keywords:** Extrarenal nephroblastomatosis, Nephrogenic rests, Paratestis, Paraspinal cord

## Abstract

**Background:**

Extrarenal nephroblastomatosis is a rare entity which occurs in retroperitoneum and inguinal region predominantly. Here we report two cases of primary extrarenal nephroblastomatosis of Han Chinese in Asian in unusual locations, one is located in testis and paratestis, and the other is paraspinal cord.

**Case presentation:**

Patient 1 was a 19-month-old boy with a hard and nodular mass adherent to the left testis in inguinal region. Patient 2 was a 9-month-old boy with a 1 × 0.7 × 0.4 cm mass in spinal canal at the midline thoracolumbar region. Histological examinations of the two patients after operations revealed extrarenall nephroblastomatosis with multiple nephrogenic foci, composed of immature glomeruli, tubules and blastemal cells.Then the patients were closely monitored without adjuvant chemotherapy, and has been alive and well without any recurrence for >6 months.

**Conclusions:**

Most nephrogenic rests remain subclinical, and thus, complete excision of the lesion with conservative treatment is recommended. Otherwise, nephrogenic rests are close associated with Wilms tumor and regular follow-up is required to ensure early detection of malignant transformation.

## Background

Extrarenal nephroblastomatosis, also called ectopic immature renal tissue (EIRT) [1–3], extrarenal nephrogenic rest (ENR) [4, 5], hamartoma with primitive renal tissue [4] and mesonephric remnant tissue [2], has been reported only rarely in the world literature. This unusual lesion is often associated with extrarenal Wilms tumor. However, the mechanism underlying the development and persistence of extrarenal nephrogenic rests remains unclear.

We retrospectively reviewed our hospital records of the past 10 years for cases of nephroblastomatosis and nephroblastoma (Wilms tumor), and identified only two cases of extrarenal nephroblastomatosis, one in the inguinal canal and the other in the vertebral canal. Here, we describe the clinical characteristics, diagnosis, treatment and embryological implications in these two cases.

## Case presentation

### Case 1

**Figure 1:**
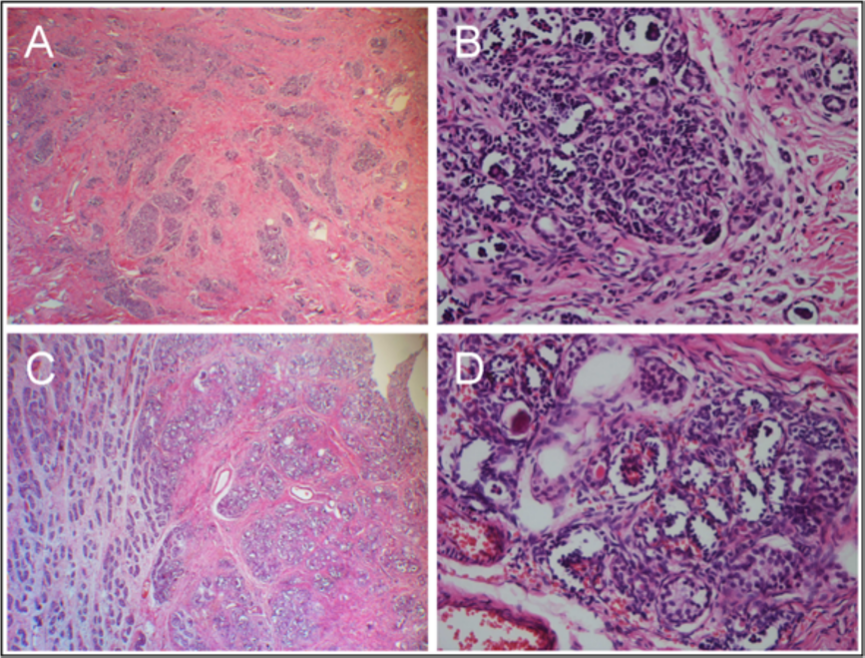
**Testicular nephrogenic rest in the inguinal canal. A**. The paratesticular mass is a complex containing fibrous tissues and multiple nephrogenic foci (hematoxylin and eosin; ×40). **B**. Nephrogenic foci are composed of immature glomeruli, tubules and blastemal cells (hematoxylin and eosin; ×400). **C**. The mass in testis contains nephrogenic foci adjacent to seminiferous tubules and rete testis (hematoxylin and eosin; ×40). **D** The lesion consistes of undifferentiated blastemal tissue, abortive glomeruli and dysplastic tubules with intervening testicular tissue (hematoxylin and eosin; ×400).

A 19-month-old boy of Han Nationality presented with an impalpable undescended left testis and the ultrasound examination revealed left cryptorchidism. Regular physical, biochemical and imaging examinations didn’t show any other abnormality then the patient underwent a left orchiopexy and the testis, which measured 0.8 × 0.5 × 0.3 cm, was found in the inguinal canal, along with a hard, undetachable, nodular mass, intimately adherent to the testis and epididymis. A portion of the mass was excised and subjected to frozen section analysis, but the result was inconclusive for a potential malignant lesion. A radical left orchiectomy was therefore performed. Examination of the resected specimen showed that the mass had invaded the testicular tissue but not the tissues surrounding the testis. Microscopic examination of paraffin-embedded tissue sections revealed that the part of the mass attached to the testis contained fibrous tissue with multiple foci of nephrogenic tissues, which were composed of immature glomeruli, tubules and blastemal cells. Within the nephrogenic tissue, mitotic figures were infrequent, and none of the mitotic figures observed were atypical (Figure 1A,B). And the other part of the lesion in the testis consisted of undifferentiated blastemal tissue, abortive glomeruli and dysplastic tubules with intervening testicular tissue. The seminiferous tubules and rete testis were adjacent to the nephrogenic rests, and the testicular development was appropriate for the patient’s age. No definitive invasive component was found in the lesion (Figure 1C,D). A multidisciplinary team, after some discussion, arrived at a final pathological diagnosis of extrarenal nephroblastomatosis, although the diagnosis of nephroblastoma could not be completely excluded. Postoperatively, conservative treatment was administered along with follow-up ultrasound examinations and clinical reviews. The patient continues to be asymptomatic for more than 6 months.

### Case 2

**Figure 2:**
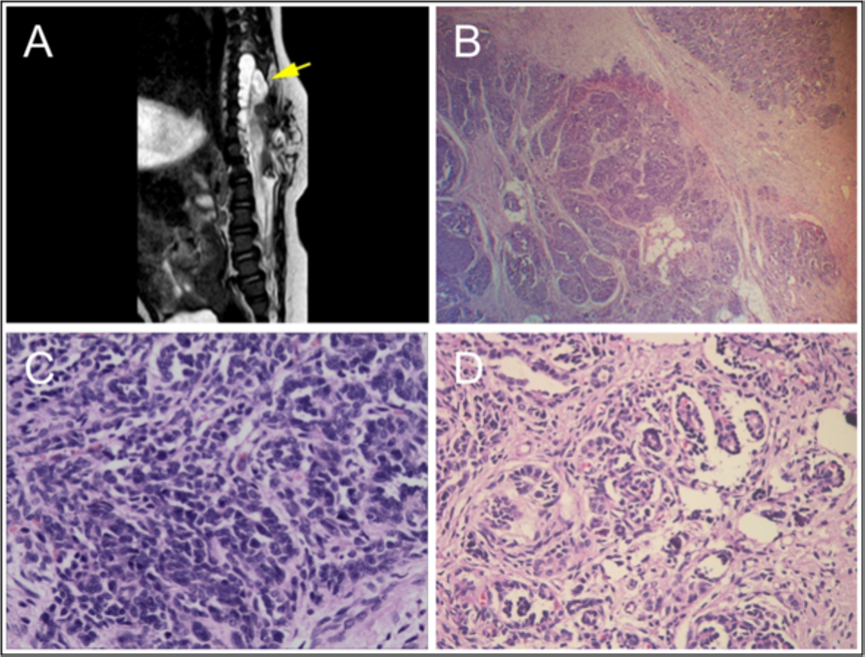
**Intraspinal nephrogenic rest in the thoracolumbar region. A**. Magnetic resonance image of a vertebral body malformation. The arrow indicates the mass. **B**. The intraspinal mass contains nervous tissues and multiple nephrogenic foci (hematoxylin and eosin; ×40). **C**. Blastemal component of the mass (hematoxylin and eosin; ×400). **D**. Nephrogenic epithelial structures (nephric tubular and glomeruli) in the mass (hematoxylin and eosin; ×400).

A 9-month-old boy of Han Nationality presented with an oval mass measuring 6 × 5 × 4 cm on his back, in the midline thoracolumbar region. The lesion was covered with normal skin. Magnetic resonance imaging (MRI) revealed a vertebral malformation with meningomyelocele, diastematomyelia and tethered cord syndrome (Figure 2A). On physical examination, knee jerk, muscular tension and muscle strength were found to be normal, and pathological reflexes were absent. No abdominal visceral or other abnormalities were detected. The patient underwent repair of meningomyelocele and diastematomyelia, and lysis of the tethered cord under general anesthesia. During the operation, we found a 1 × 0.7 × 0.4 cm mass just adherent to the surface of the spinal cord at the T7-L1 level. On pathological examination, this mass was found to consist of a blastemal component and nephrogenic epithelial tubular and rudimentary glomeruloid structures, resembling immature ectopic nephrogenic tissue, with occasional neurogenic tissue and dystrophic calcifications. Mitoses were rare, and none of those observed were atypical (Figure 2B,C,D). The pathological diagnosis was extrarenal nephroblastomatosis without evidence of a neoplastic process. The lesion appeared to have been completely excised. Multiple pediatric experts recommended close observation without adjuvant chemotherapy. The patient has been alive and well without any evidence of recurrence or malignant transformation for over 6 months.

## Discussion

Kidney development, which is also called nephrogenesis, is a complex process involving tissues with two distinct embryological origins: nephrogenic and ductogenic [6]. Nephrogenic tissue develops from the intermediate mesoderm and proceeds through a series of three successive phases: pronephros, mesonephros and metanephros. This process normally ceases after 36 weeks of gestation, at which time, the metanephric blastema in the kidney disappears [7]. Occasionally, the nephrogenic blastema fails to mature into normal renal parenchyma, and the persistent blastemal tissue is then referred to as nephrogenic rests, which may undergo oncogenic mutation to form a malignant embryonal tumor known as nephroblastoma. On histological examination, nephrogenic rests usually appear as aggregates of abnormally persistent embryonal nephroblastic tissue with small clusters of blastemal cells, tubules and occasional glomeruli, with a variable amount of admixed fibrous stroma. Nephrogenic rests can be unifocal, multifocal or diffuse. The term nephroblastomatosis is used to refer to diffuse or multifocal nephrogenic rests or their derivatives [8]. Nephrogenic rests can be subclassified into perilobar nephrogenic rests and intralobar nephrogenic rests, according to their location in relation to the renal lobe on histological examination. Occasionally, nephrogenic rests are found in ectopic sites, such as the inguinal canal [5], lumbosacral area [3, 9–12], adrenal gland [13], thorax [14, 15], colon [16] and heart [17]. There are two possible distinct fates for nephrogenic rests: to be an isolated developmental abnormality, or the early stage of a neoplastic process, with the latter being more clinically significant. Most nephrogenic rests become dormant, mature or spontaneously regress. Some undergo hyperplastic overgrowth, which is hypothesized to be an intermediate, pre-neoplastic stage in the process of tumorigenesis. Only a small number undergo a neoplastic induction to transform into Wilms tumor, which is the most common malignant renal neoplasm in children [18].

Differential diagnosis between extrarenal nephroblastomatosis and extrarenal nephroblastoma is mandatory when ectopic immature renal tissue is found. Sometimes it is difficult to distinguish ectopic nephrogenic rests from Wilms tumor on histology, and it is especially difficult to distinguish between a proliferative nephrogenic rest and a small Wilms tumor [19]. Generally, nephroblastoma tends to form round nodules enclosed in a fibrous pseudocapsule owing to its rapidly expansile growth. On histological examination, the lesion comprises nephrogenic tissues, such as blastemal, epithelial (tubular and glomeruloid) and mesenchymal elements. The distinguishing characteristic of nephroblastoma is frank atypia including disordered structures, atypical mitoses and marked pleomorphism. Otherwise, approximately 5% of Wilms tumors show anaplastic cells, which is a typical characteristic of malignant, indicating poor prognosis [20, 21]. In contrast, the lesion in nephroblastomatosis usually consists of small multiple microscopic nests and islets. Mitoses are usually sparse, except in proliferative nodules, which exhibit high mitotic rates and moderate pleomorphism, but no obvious atypia. Proliferative nephrogenic rests invariably show striking enlargement, but lack the peritumoral pseudocapsule characteristic of Wilms tumor.

In both our patients, the possible differential diagnoses included teratoma, a neoplasm originating from testicular tissue, metastasis and malignancies other than Wilms tumor. The nephrogenic epithelial elements (glomeruli, tubules) in the two cases had differentiated to a rather advanced degree, and rare mitoses with no atypia were found scattered within the dense fibrous tissue, indicating a benign neoplasm. The diagnosis of teratoma was excluded due to the lack of other teratomatous non-nephrogenic tissues.

The ectopic nephrogenic rests were thought to have originated from mesonephric or metanephric tissue. In the few published studies on this topic, ectopic nephrogenic rests have been reported predominantly in the retroperitoneum and inguinal region [22]. In our first patient, the mass was attached to the testis, which is consistent with an nephrogenic rest arising from mesonephric tissue, because the mesonephros is associated with the developing gonad, embryologically [23]. There have also been several case reports of intraspinal nephrogenic rests located in the lumbosacral area and frequently associated with spinal dysraphism [3, 10]. This peculiar association of spinal abnormalities with nephrogenic rests in the lumbosacral region, where the metanephros comes closest to the spinal cord [24], supports the hypothesis that neural tube abnormalities interfere with the migration of renal tissue and thereby result in ectopic nephrogenic rests [4, 12]. However, in our second patient, the lesion was located in the lower thoracic region, indicating that nephrogenic remnants may be trapped between the dura and the developing spinal cord early during nephrogenesis.

## Conclusions

From the scarce literature available on this subject, we conclude that most nephrogenic rests can be expected to remain subclinical, and thus, complete excision of the lesion with conservative treatment is recommended. However, it is reported that approximately 40% Wilms tumors were associated with nephrogenic rests [19]. Owing to the close association between renal nephrogenic rests and Wilms tumor, regular follow-up is required for such lesions until the period of risk has ended. This ensures the detection of malignant transformation at an early stage [25, 26].

## Consent

Written informed consent was obtained from the patient’s parents for publication of this case report and any accompanying images. A copy of the written consents is available for review by the Editor of this journal.

### Ethics

The materials and data of the patients have been performed in accordance with the Declaration of Helsink and have been approved by an appropriate ethics committee of Soochow University Affiliated Children’s Hospital.
